# Macronutrient Composition of the Diet and Prospective Weight Change in Participants of the EPIC-PANACEA Study

**DOI:** 10.1371/journal.pone.0057300

**Published:** 2013-03-05

**Authors:** Anne-Claire Vergnaud, Teresa Norat, Traci Mouw, Dora Romaguera, Anne M. May, H. Bas Bueno-de-Mesquita, Daphne van der A, Antonio Agudo, Nicholas Wareham, Kay-Tee Khaw, Isabelle Romieu, Heinz Freisling, Nadia Slimani, Florence Perquier, Marie-Christine Boutron-Ruault, Françoise Clavel-Chapelon, Domenico Palli, Franco Berrino, Amalia Mattiello, Rosario Tumino, Fulvio Ricceri, Laudina Rodríguez, Esther Molina-Montes, Pilar Amiano, Aurelio Barricarte, Maria-Dolores Chirlaque, Francesca L. Crowe, Philippos Orfanos, Androniki Naska, Antonia Trichopoulou, Birgit Teucher, Rudolf Kaaks, Heiner Boeing, Brian Buijsse, Ingeged Johansson, Göran Hallmans, Isabel Drake, Emily Sonestedt, Marianne Uhre Jakobsen, Kim Overvad, Anne Tjønneland, Jytte Halkjær, Guri Skeie, Tonje Braaten, Eiliv Lund, Elio Riboli, Petra H. M. Peeters

**Affiliations:** 1 Department of Epidemiology & Public Health, Imperial College London, London, United Kingdom; 2 Julius Centre for Health Sciences and Primary Care, University Medical Centre Utrecht, Utrecht, The Netherlands; 3 National Institute for Public Health and the Environment (RIVM), Bilthoven, The Netherlands; 4 Department of Gastroenterology and Hepatology, University Medical Centre, Utrecht, The Netherlands; 5 Unit of Nutrition, Environment and Cancer, Catalan Institute of Oncology, IDIBELL, Barcelona, Spain; 6 Medical Research Council, Epidemiology Unit, Institute of Metabolic Science, Cambridge, United Kingdom; 7 Department of Public Health and Primary Care, University of Cambridge, Cambridge, United Kingdom; 8 International Agency for Research on Cancer (IARC-WHO), Lyon, France; 9 Institut National de la Santé et de la Recherche Médicale (INSERM), ERI 20, EA 4045, Villejuif, France; 10 Institut Gustave Roussy, Villejuif, France; 11 Molecular and Nutritional Epidemiology Unit, CSPO-Scientific Institute of Tuscany, Florence, Italy; 12 Epidemiology and Prevention Unit, IRCCS Foundation, National Cancer Institute, Milan, Italy; 13 Department of Clinical and Experimental Medicine, Federico II University, Naples, Italy; 14 Cancer Registry and Histopathology Unit, “Civile M.p.Arezzo” Hospital, Ragusa, Italy; 15 Human Genetics Foundation, HuGeF, Turin, Italy; 16 Public Health and Participation Directorate, Health and Health Care Services Council, Asturias, Spain; 17 Andalusian School of Public Health, Granada, Spain; 18 Public Health Department of Gipuzkoa, Basque Regional Health Department, San Sebastian, Spain and Biodonostia; 19 Navarre Public Health Institute, Pamplona, Spain; 20 Consortium for Biomedical Research in Epidemiology and Public Health (CIBER Epidemiología y Salud Pública-CIBERESP), Spain; 21 Department of Epidemiology, Regional Health Authority, Murcia, Spain; 22 Cancer Epidemiology Unit Nuffield Department of Clinical Medicine University of Oxford, Oxford, United Kingdom; 23 WHO Collaborating Center for Food and Nutrition Policies, Department of Hygiene, Epidemiology and Medical Statistics, University of Athens Medical School, Athens, Greece; 24 Hellenic Health Foundation, Athens, Greece; 25 Division of Clinical Epidemiology, German Cancer Research Center, Heidelberg, Germany; 26 Department of Epidemiology, German Institute of Human Nutrition, Potsdam-Rehbruecke, Germany; 27 Department of Odontology, Umeå University, Umeå, Sweden; 28 Department of Public Health and Clinical Medicine, Nutritional Research, Umeå University, Umeå, Sweden; 29 Department of Clinical Sciences in Malmö, Nutrition Epidemiology, Lund University, Malmö, Sweden; 30 Department of Epidemiology School of Public Health Aarhus University, Aarhus, Denmark; 31 Department of Cardiology Center for Cardiovascular Research Aalborg Hospital, Aarhus University Hospital, Aalborg, Denmark; 32 Danish Cancer Society Research Center, Copenhagen, Denmark; 33 Institute of Community Medicine, University of Tromsø, Tromsø, Norway; Wageningen University, The Netherlands

## Abstract

**Background:**

The effect of the macronutrient composition of the usual diet on long term weight maintenance remains controversial.

**Methods:**

373,803 subjects aged 25–70 years were recruited in 10 European countries (1992–2000) in the PANACEA project of the EPIC cohort. Diet was assessed at baseline using country-specific validated questionnaires and weight and height were measured at baseline and self-reported at follow-up in most centers. The association between weight change after 5 years of follow-up and the iso-energetic replacement of 5% of energy from one macronutrient by 5% of energy from another macronutrient was assessed using multivariate linear mixed-models. The risk of becoming overweight or obese after 5 years was investigated using multivariate Poisson regressions stratified according to initial Body Mass Index.

**Results:**

A higher proportion of energy from fat at the expense of carbohydrates was not significantly associated with weight change after 5 years. However, a higher proportion of energy from protein at the expense of fat was positively associated with weight gain. A higher proportion of energy from protein at the expense of carbohydrates was also positively associated with weight gain, especially when carbohydrates were rich in fibre. The association between percentage of energy from protein and weight change was slightly stronger in overweight participants, former smokers, participants ≥60 years old, participants underreporting their energy intake and participants with a prudent dietary pattern. Compared to diets with no more than 14% of energy from protein, diets with more than 22% of energy from protein were associated with a 23–24% higher risk of becoming overweight or obese in normal weight and overweight subjects at baseline.

**Conclusion:**

Our results show that participants consuming an amount of protein above the protein intake recommended by the American Diabetes Association may experience a higher risk of becoming overweight or obese during adult life.

## Introduction

Obesity is a growing epidemic now affecting developing and industrialized countries alike [1]. It is associated with an increased risk for several diseases including cardiovascular diseases, diabetes and several cancers [1] and the importance of its prevention and treatment is widely acknowledged.

A positive balance between energy intake and energy expenditure is necessary to gain weight. In both the United States [2] and Europe [3], the food energy supply has increased steadily from the 70's to 2000. Although leisure-time physical activity seems to have increased, occupational and transport activity have declined substantially suggesting an increase in the total burden of physical inactivity [4], [5]. In addition, macronutrient composition, especially a high proportion of carbohydrates, has also been suggested as an important determinant in the obesity epidemic since the relative proportion of energy from each macronutrient has changed in the United States in conjunction with the increase in energy intake [6], [7]. Protein intake stayed relatively stable (14% to 15% of energy intake) but total fat decreased from 36% to 33% of energy while carbohydrates increased from 44% to 50% of energy. The reverse was observed in Europe where the proportion of fat increased at the expense of carbohydrates [3]. In addition, the amount of protein from animal sources increased in some countries [3]. The Food and Agriculture Organisation (FAO) also reported an increased in the consumption of meat in the United States, Europe and many developing countries [8]. Observational studies investigating the associations between diet composition and weight gain or obesity have also been inconclusive so far [9].

Therefore, the consequences of the macronutrient composition of the usual diet in free living subjects still remain controversial, especially as results from the few long term prospective studies [10]–[14] seem often in contradiction with some short term intervention studies showing the efficacy of low carbohydrate/high protein diets on weight loss [15]–[17]. Weight maintenance through adult life in non-dieters corresponds to a very different metabolic state than weight maintenance following rapid weight loss in obese subjects [18] and can only be investigated using long-term observational cohort studies. However, previous findings may have been limited by insufficient statistical power due to small sample size or homogeneous dietary intake [9].

The European Prospective Investigation into Cancer and Nutrition – Physical Activity, Nutrition, Alcohol, Cessation of Smoking, Eating Out of Home and Obesity (EPIC-PANACEA) study recruited half a million participants from 10 different European countries with heterogeneous dietary patterns and obesity prevalence. It constitutes a unique opportunity to explore the relation between diet and obesity. The objective of the present study is to investigate the relationship between the macronutrient composition of the usual diet and weight change after 5 years on average, taking into account lifestyle factors.

## Methods

### Study population

EPIC is a multi-center, prospective cohort study investigating the role of metabolic, dietary, lifestyle, and environmental factors in the development of cancer and other chronic diseases. Briefly, between 1992 and 2000, 521,448 volunteers aged between 25 and 70 years were recruited in 23 centers from 10 European countries (Denmark, France, Germany, Greece, Italy, The Netherlands, Norway, Spain, Sweden, and the United Kingdom). In France, Norway, Utrecht (The Netherlands) and Naples (Italy), only women were included. Individuals were selected from the general population except in the French cohort, based on state-school employees and the Utrecht cohort, based on women who underwent breast cancer screening. Furthermore, a large portion of the centers of Spain and Italy were blood donors. Participant eligibility within each center was based essentially on geographic or administrative boundaries. Approval for this study was obtained from the ethical review boards of the International Agency for Research on Cancer and from all local institutions. Details of the recruitment, study design and data collection have been previously published [19], [20].

For the present study, we excluded 23,713 individuals with missing dietary and non-dietary questionnaires, missing data on weight and height at baseline, extreme or implausible anthropometric values, pregnant women, and those in the top and bottom 1% of the ratio between energy intake to estimated energy requirement. We further excluded 121,866 subjects with missing weight at follow-up and 2,066 subjects with extreme or implausible weight changes. Thus, 373,803 subjects (103,455 men and 270,348 women) were included in the present analysis (more details have been previously published [21]).

### Assessment of anthropometric measures and weight change

Two weight measures were available for each participant: one measure at baseline (between 1992 and 2000 depending of the center) and one at the latest follow-up (after 5 years on average; minimum: 2 years for Heidelberg; maximum: 11 years for Varese). At baseline, body weight and height were measured in most centers using similar, standardized procedures. The exceptions were France, Norway and the health conscious group of the Oxford center (United Kingdom) where self-reported anthropometric values were collected. At follow-up, self-reported weight were obtained in all centers, except in Norfolk (United Kingdom) and Doetinchem (The Netherlands) where weight was measured. Assessment of self-reported weight was conducted through mailed questionnaires, with several exceptions: Spain and Greece completed the questionnaire on the phone and Varese used a combination of postal survey and telephone interview. The accuracy of all self-reported anthropometric measures was improved with the use of prediction equations derived from subjects with both measured and self-reported measures [22]. As the follow-up times differ by center, our main outcome is the 5y weight change (g/5y) (*i.e.* (weight at follow-up – weight at baseline) ×5/years of follow-up). Recent findings from the EPIC-Potsdam study based on 5 measurements of weight suggest that weight gain can be reasonably well approximated by a straight line over a follow-up period of 8 years on the population-level [23].

### Dietary assessment

Usual dietary intake at baseline was measured using country-specific validated questionnaires developed to capture geographical specificity of diet. Most centers adopted a self-administered quantitative dietary questionnaire of 88–266 food items [20]. Semi-quantitative food frequency questionnaires were used in Denmark, Norway, Naples and Umea and interviewer-administered dietary questionnaires were used in Ragusa, Naples, Spain and Greece. Nutrient intakes were calculated using the ‘EPIC Nutrient DataBase’, a standardized food-composition table [24]. In order to adjust for possible systematic under- or overestimation in dietary intake measures, a dietary calibration study was conducted [25] using a random sample of about 36,900 men and women that completed an additional standardized computerized 24-hour dietary recall (EPIC-Soft®, IARC, Rhone-Alpes, Lyon, France). The correlation coefficients between the urinary nitrogen and the dietary nitrogen were 0.53 and 0.86 when estimated from the food frequency questionnaire and the 24-hour dietary recall respectively [26].

### Assessment of other covariates

Lifestyle and health factors (tobacco smoking, educational attainment, physical activity and history of previous illness) were collected by questionnaires at baseline [20]. For smoking status only, a second measure was collected by questionnaires during follow-up at the same time as the anthropometric measures. This permitted to take into account smoking status modification during follow-up in a sensitivity analysis.

### Statistical analyses

We first examined the distribution of population's main characteristics according to the sex-specific tertiles of the percentage of energy coming from each macronutrient using ANCOVA or chi-square tests as appropriate. Means adjusted for center are presented for continuous variables. The 5y weight gain means were further adjusted for initial BMI.

The association between macronutrient and 5y weight change (g/5y) was investigated using 2-levels (individuals within centers) mixed effects linear regression models with each macronutrient on a continuous scale. Sex, age, initial BMI (kg/m^2^), follow-up time (years), educational attainment (primary school, technical school, secondary school and university degree; categorically), physical activity index (combination of occupational physical activity, cycling and sport activities in four categories: inactive, moderately inactive, moderately active and active [27]), smoking status at baseline (never, former and current smoker; categorically) and a categorical variable indicating plausibility of energy intake reporting, were considered as confounding factors. Participants were classified as under-reporters (ratio of reported energy intake to predicted basal metabolic rate (EI:BMR) <1.14), plausible reporters (EI:BMR = 1.14–2.1) or over-reporters (EI:BMR>2.1) using cut-off points proposed by Goldberg [28]. Subjects with missing value for educational attainment (n = 14,092), physical activity index (n = 43,275) or smoking status (n = 7,708) were classified as a separated category. Intercept and macronutrient slope were entered as both fixed and random effects and the confounding factors were entered as fixed effects in the model.

Several multivariate substitution models were performed [29] to estimate the weight change associated with the iso-energetic replacement of 5% of energy from one macronutrient by 5% of energy from another macronutrient. For example, for an iso-energetic replacement of 5% of energy from carbohydrates by 5% of energy from protein, the percentages of energy from protein and from fat, as well as total energy from non alcohol sources (kcal) and energy from alcohol sources (kcal) were further included as independent variables (fixed effects). The interpretation of the protein parameter is the weight change associated with a 5% higher proportion of protein, while keeping the percentages of fat, and energy constant, i.e., at the expense of carbohydrates, which is not included in the model. Substitution models were performed for each macronutrient (protein, fat and carbohydrates) as well as each macronutrient sub-type (animal and plant protein, animal and plant fat, sugar and starch). Substitution models were chosen to distinguish as best as possible the effect of macronutrient composition alteration from the effect of energy intake modification.

Analyses were also performed using calibrated dietary data obtained from country- and sex-specific calibration models as previously described [21], [30]. For each macronutrient separately, the 24-hour dietary values were regressed on the dietary values obtained from the main dietary questionnaire, adjusting for age, BMI at baseline, total energy from non alcohol sources, energy from alcohol sources and study center. Data were weighted by the day of the week and the season of the year in which the 24-hour dietary recall was collected. The standard error of the coefficient was estimated using bootstrap sampling (10 loops). Statistical significance was judged at α<0.05.

We evaluated whether the effect associated with a change in the carbohydrates proportion differed according to the glycemic index of the diet [31] by including interaction terms between each macronutrient proportion (fat or protein) and the median of the glycemic index (0: below the median; 1: above the median). Similar analyses were performed to evaluate whether the results differed according to the fiber intake median.

In order to address whether the associations could be modified by dietary mis-reporting or change in diet, we conducted sensitivity analyses excluding participants with chronic diseases at baseline (heart disease, stroke, diabetes mellitus, hypertension, hyperlipidemia and/or cancer, n = 76,077), those likely to misreport energy intake (n = 121,425) [28] and subjects with incident cancer (n = 9,144) or smoking status modification during follow-up (n = 24,051).

We explored potential effect modification by age, BMI category at baseline, smoking status, level of education, physical activity and dietary pattern by including interaction terms between each variable and percentage of energy from macronutrient in the models. Dietary patterns were derived from maximum likelihood factor analysis as previously described [21]. The “prudent pattern” distinguished participants with high intakes of vegetables, legumes, fruits, pasta & rice and vegetable oils, from those with high intakes of processed meat, potatoes, margarines, coffee & tea and beer & cider. Center-specific associations were investigated with multivariate Generalized Linear Models adjusted for energy from non alcohol sources, energy from alcohol sources and confounding factors previously described.

We studied the association between categories of energy from protein and the risk of becoming overweight, obese, or morbidly obese after 5-y of follow-up. The modified Poisson regression approach of Zou [32] was used to calculate the relative risks (RR) of becoming overweight, obese or morbidly obese according to the percentage of energy from protein. We used the high-protein diet cut-off point from the American Diabetes Association (>20% of energy from protein) and further categorized participants by 2% increases (≤14, 14.1–16, 16.1–18, 18.1–20, 20.1–22, >22%) to determine our categories. Analyses were stratified by initial BMI categories (<25: normal weight, 25≤BMI<30: overweight and 30≤BMI<40 kg/m^2^: obese). Subjects morbidly obese at baseline (BMI≥40 kg/m^2^) were excluded (n = 1,957). Relative risks were adjusted for energy from non alcohol sources, energy from alcohol sources, center and confounding factors previously described. The BMI after 5-y was calculated from the 5-y weight change and their baseline height.

All statistical analyses were performed with SAS 9.2 (Cary, NC, USA) or STATA 10.0 (College Station TX).

### Ethical approval

Approval for this study was obtained from the ethical review boards of the International Agency for Research on Cancer and from all local institutions where subjects had been recruited for the EPIC study.

## Results

The average percentages of energy from each macronutrient were 43.8%, 35.4% and 17.0% for carbohydrates, fat and protein respectively. Percentage of energy from carbohydrates was negatively correlated to percentages of energy from fat (r = −0.68) and to a lesser extent to protein (r = −0.27). Percentage of energy from protein was not correlated to percentage of energy from fat (r = −0.04). Characteristics of the population according to sex and percentages of energy from each macronutrient are presented in **Table 1**. Compared to subjects in the first tertile of energy from carbohydrates, subjects in the third tertile were slightly older, more often physically active and current smoker, had a lower BMI at baseline, a lower weight gain during follow-up and reported a slightly lower energy intake. In men, there was a higher proportion of participants with a university degree but not in women. Compared to subjects in the first tertile of energy from protein, subjects in the third tertile were slightly older, had a higher BMI at baseline, a higher weight gain during follow-up and reported a lower energy intake. There was a lower proportion of participants with a university degree and a higher proportion of current smoker. In men, they were more often physically active but not in women. Compared to subjects in the first tertile of energy from fat, subjects in the third tertile were slightly younger and reported higher energy intake. They were less often physically active and more often smokers. In men, they had less often a university degree but not in women. Initial BMI was not different according to the tertiles of energy coming from fat in neither men nor women. A higher weight gain in the last tertile of energy from fat compared to the first one was observed in women but not in men.

**Table 1:** Characteristics of the population according to gender and percentage of energy from carbohydrates, protein and fat (n = 373,803).

	Tertiles of percentage of energy from carbohydrates			Tertiles of percentage of energy from protein			Tertiles of percentage of energy from fat		
	1	2	3	1	2	3	1	2	3
**Men**									
% carbohydrates, median [min; max]	35.5 [5.3; 39.2]	42.4 [39.3; 45.5]	49.3 [45.6; 84.4]	44.7 [12.7; 84.4]	42.4 [15.9; 71.3]	40.3 [5.3; 68.7]	47.1 [14.8; 84.4]	43.5 [5.3; 58.0]	38.3 [5.9; 52.6]
% protein, median [min; max]	16.8 [4.2; 39.5]	16.2 [5.8; 34.1]	15.1 [5.8; 32.7]	13.5 [4.2; 14.9]	16.0 [15.0; 17.3]	19.0 [17.4; 39.6]	16.0 [4.2; 39.5]	16.2 [6.2; 37.1]	15.9 [6.6; 38.3]
% fat, median [min; max]	38.8 [0.6; 65.6]	36.1 [10.8; 52.3]	31.6 [8.3; 44.8]	35.0 [0.6; 65.6]	35.3 [11.2; 61.5]	34.8 [8.3; 65.2]	29.3 [0.6; 32.5]	35.0 [32.6; 37.6]	41.0 [37.7; 65.6]
Age (years) 1	51.5±0.1	51.7±0.1	52.2±0.1	51.6±0.1	51.7±0.1	52.0±0.1	52.4±0.1	51.7±0.1	51.2±0.1
Initial BMI (kg/m^2^) 1	27.2±0.0	26.7±0.0	26.4±0.0	26.2±0.0	26.6±0.0	27.2±0.0	26.8±0.0	26.7±0.0	26.7±0.0
5y weight gain (g/5y) ^2^	2223±31	2257±29	2142±30	2064±32	2155±29	2344±29	2244±29	2147±29	2229±31
Total energy (kcal) 1	2491±3.9	2453±3.6	2416±3.8	2621±4.0	2497±3.7	2303±3.6	2353±3.6	2458±3.6	2566±3.9
Glycemic index^1^	57.1±0.0	57.2±0.0	57.4±0.0	57.8±0.0	57.4±0.0	56.8±0.0	57.3±0.0	57.3±0.0	57.2±0.0
“Prudent” dietary pattern score^1^	−0.17±0.00	−0.09±0.00	0.05±0.00	−0.09±0.00	−0.08±0.00	−0.05±0.00	−0.07±0.00	−0.08±0.00	−0.07±0.00
University degree and longer (%)	25.1	28.2	28.5	30.8	27.3	23.8	28.3	28.8	24.7
Physically active subjects (%)	21.6	23.8	23.2	21.2	23.1	24.2	25.1	23.3	20.2
Tobacco status (%)									
Never smoker	24.4	33.1	42.1	35.7	33.1	30.8	33.2	34.6	31.7
Former smoker	35.6	37.8	37.3	36.4	37.0	37.2	39.8	36.9	34.0
Current smoker	38.6	27.9	19.5	26.4	28.5	31.1	26.3	27.5	32.2
Previous illness (%)	8.7	7.4	7.8	6.3	7.4	10.2	8.7	7.2	8.1
**Women**									
% carbohydrates, median [min; max]	37.7 [8.6; 41.4]	44.4 [41.5; 47.3]	50.9 [47.4; 82.8]	46.5 [11.7; 82.8]	44.5 [12.3; 71.1]	42.3 [8.6; 70.2]	50.3 [9.5; 82.8]	44.9 [11.7; 59.4]	39.1 [8.6; 53.3]
% protein, median [min; max]	17.9 [5.4; 45.6]	17.2 [6.2; 32.9]	16.1 [5.8; 37.7]	14.4 [5.4; 15.8]	17.1 [15.9; 18.4]	20.1 [18.4; 45.6]	17.2 [5.4; 42.1]	17.1 [6.7; 45.6]	16.8 [5.8; 40.1]
% fat, median [min; max]	40.5 [10.1; 71.6]	35.8 [10.6; 49.8]	30.6 [5.1; 43.5]	35.7 [5.1; 71.6]	35.3 [10.3; 61.7]	34.9 [9.5; 68.2]	29.6 [5.1; 32.8]	35.3 [32.9; 37.9]	41.3 [38.0; 71.6]
Age (years) 1	50.09±0.0	50.86±0.0	51.40±0.0	50.14±0.0	50.83±0.0	51.29±0.0	51.58±0.0	50.74±0.0	50.02±0.0
Initial BMI (kg/m^2^) 1	25.75±0.0	25.53±0.0	25.37±0.0	24.75±0.0	25.42±0.0	26.30±0.0	25.55±0.0	25.52±0.0	25.58±0.0
5y weight gain (g/5y) ^2^	2132±19	1983±18	1923±19	1815±20	1895±18	2282±19	1983±19	1953±18	2101±19
Total energy (kcal) 1	1952±2.0	1963±1.9	1925±2.0	2054±2.1	1986±1.9	1825±1.9	1860±2.0	1963±1.9	2021±2.0
Glycemic index^1^	55.1±0.0	55.9±0.0	56.2±0.0	56.4±0.0	56.0±0.0	55.0±0.0	55.8±0.0	55.9±0.0	55.4±0.0
“Prudent” dietary pattern score^1^	0.14±0.00	0.15±0.00	0.24±0.00	0.19±0.00	0.16±0.00	0.18±0.00	0.24±0.00	0.14±0.00	0.15±0.00
University degree and longer (%)	24.6	22.2	22.9	27.1	23.1	19.5	23.3	23.5	23.0
Physically active subjects (%)	11.8	13.8	14.4	14.7	13.4	11.8	15.2	13.9	10.9
Tobacco status (%)									
Never smoker	54.2	57.4	60.8	58.2	58.1	56.1	56.2	57.0	59.2
Former smoker	21.3	23.1	23.0	22.1	22.3	23.0	25.5	22.9	19.1
Current smoker	21.7	17.4	14.0	17.7	17.1	18.3	16.2	18.0	18.9
Previous illness (%)	8.3	7.3	7.5	7.2	7.4	8.4	7.7	7.2	8.2

Adjusted means (standard error) are presented for continuous variables and percentages are presented for categorical variables. All P-values are <0.05.

1 Adjusted for center ^2^ Adjusted for center and initial body mass index.

Adjusted 5-y weight change (g/5y) for the iso-energetic replacement of 5% of energy from one macronutrient by 5% of energy from another macronutrient are presented in **Table 2**. A 5% higher proportion of fat at the expense of carbohydrates was not associated with weight change in men and women. Similar findings were observed for plant fat. The substitution of carbohydrate by animal fat was weakly negatively associated with weight gain in the uncalibrated model but this association disappeared in the calibrated model. Similarly, the positive association observed between weight gain and the substitution of animal fat by plant fat in the uncalibrated model was no longer significant in the calibrated model. A 5% higher proportion of protein at the expense of carbohydrates was associated with a 247g weight gain in men (95% CI = (160,334)) and a 388g weight gain (296,480) in women after 5 years. Similar associations were observed when protein was increased at the expense of fat (β (95% CI) = 275 (184,366) in men and 397 (303,491) in women). These associations were strengthened when using calibrated data and were observed for both animal protein and plant protein (in models where animal protein and plant protein were adjusted for each other only). The substitution of animal protein by plant protein and the substitution of sugar by starch were not consistently associated with weight change in uncalibrated and calibrated data. Excluding subjects with previous diseases, implausible energy reporters according to the Goldberg criteria or those with cancer or smoking status modification during follow-up did not substantially change the results (data not presented).

**Table 2:** Adjusted 5y weight change (in g/5y) for the iso-energetic increase of 5% of energy from one macronutrient (↑) at the expense of 5% of energy from another macronutrient (↓) according to gender before and after calibration (n = 373,803).^1^

Type of macronutrient	Men				Women			
Substitution	Uncalibrated data		Calibrated data ^2^		Uncalibrated data		Calibrated data ^2^	
	β (95% CI)	P	β (95% CI)	P	β (95% CI)	P	β (95% CI)	P
**Fat↑**								
Non fat energy↓	−28 (−66,9)	0.14	−21 (−120,77)	0.68	−25 (−72,22)	0.31	−94 (−362,175)	0.49
Carbohydrate↓ ^3^	−29 (−66,9)	0.13	−17 (−110,77)	0.73	−21 (−63,21)	0.33	−105 (−331,120)	0.36
Protein↓^4^	−181 (−245, −117)	<0.0001	−283 (−473, −93)	0.003	−339 (−393, −285)	<0.0001	−772 (−1064, −480)	<0.0001
**Animal fat↑**								
Non animal fat energy↓	−11 (−41,18)	0.45	−23 (−104,59)	0.58	−10 (−43,23)	0.56	−55 (−294,183)	0.65
Carbohydrate↓ ^3,6,7^	−54 (−88, −19)	0.002	−87 (−212,39)	0.18	−65 (−94, −36)	<0.0001	−236 (−489,18)	0.07
Protein↓^4,6,7^	−212 (−282, −142)	<0.0001	−258 (−455, −61)	0.01	−444 (−493, −395)	<0.0001	−817 (−1165, −469)	<0.0001
**Plant fat↑**								
Non plant fat energy↓	−6 (−45,34)	0.78	−21 (−162,119)	0.77	3 (−57,62)	0.93	−1 (−259,257)	1.00
Carbohydrate↓ ^3,7,8^	−9 (−54,36)	0.69	−11 (−189,166)	0.90	45 (−15,105)	0.14	104 (−61,270)	0.22
Protein↓^4,7,8^	−183 (−251, −115)	<0.0001	−295 (−507, −84)	0.006	−352 (−420, −285)	<0.0001	−524 (−841, −207)	0.001
Animal fat↓^3,5,7^	42 (−2,87)	0.06	75 (−115,264)	0.44	112 (51,172)	0.0003	198 (−148,544)	0.26
**Protein↑**								
Non protein energy↓	247 (159,334)	<0.0001	401 (35,766)	0.03	388 (296,480)	<0.0001	608 (129,1088)	0.01
Carbohydrate↓ ^4^	247 (160,334)	<0.0001	399 (49,749)	0.02	388 (296,480)	<0.0001	609 (10,1207)	0.05
Fat↓ ^5^	275 (184,366)	<0.0001	415 (61,770)	0.02	397 (303,491)	<0.0001	605 (131,1078)	0.01
**Animal protein↑**								
Non animal protein↓	173 (84,263)	0.0001	224 (−128,575)	0.21	295 (211,380)	<0.0001	436 (−17,889)	0.06
Carbohydrate↓ ^4,9,10^	251 (159,343)	<0.0001	482 (95,869)	0.01	385 (295,474)	<0.0001	685 (250,1120)	0.002
Fat↓ ^5,9,10^	270 (170,365)	<0.0001	435 (105,770)	0.010	399 (307,490)	<0.0001	699 (254,1144)	0.002
**Plant protein↑**								
Non plant protein energy↓	114 (−56,285)	0.19	343 (−51,737)	0.09	−74 (−223,76)	0.33	−3 (−855,850)	1.00
Carbohydrate↓ ^4,10,11^	363 (165,561)	0.0003	1120 (644,1595)	<0.0001	418 (271,566)	<0.0001	1306 (321,2291)	0.009
Fat↓ ^5,10,11^	375 (185,565)	0.0001	1120 (650,1590)	<0.0001	399 (257,542)	<0.0001	1306 (375,2237)	0.006
Animal protein↓ ^4,5,10^	158 (−31,346)	0.10	824 (381,1267)	0.0003	3 (−143,148)	0.97	537 (−438,1511)	0.28
**Carbohydrate↑**								
Non carbohydrate energy↓	−25 (−69,18)	0.25	−54 (−135,27)	0.19	−39 (−83,4)	0.07	−72 (−209,65)	0.30
Fat↓ ^3^	21 (−25,67)	0.37	20 (−58,98)	0.62	41 (−3,84)	0.06	102 (−52,256)	0.19
Protein↓ ^4^	−174 (−240, −109)	<0.0001	−309 (−487, −130)	0.0007	−307 (−359, −254)	<0.0001	−600 (−823, −377)	<0.0001
**Sugar↑**								
Non sugar energy↓	−40 (−91,12)	0.13	−114 (−222, −5)	0.04	0 (−31,31)	0.99	15 (−107,137)	0.81
Fat↓ ^3,12^	12 (−44,68)	0.66	−38 (−154,77)	0.52	43 (12,74)	0.007	83 (−39,206)	0.18
Protein↓ ^4,12^	−179 (−248, −109)	<0.0001	−307 (−442, −172)	<0.0001	−310 (−352, −268)	<0.0001	−484 (−641, −327)	<0.0001
**Starch↑**								
Non starch energy↓	6 (−38,49)	0.79	69 (−34,172)	0.19	−50 (−89, −11)	0.01	−176 (−303, −49)	0.007
Fat↓ ^3,13^	22 (−25,68)	0.36	71 (−65,207)	0.31	3 (−36,41)	0.89	−58 (−219,104)	0.48
Protein↓ ^4,13^	−158 (−223, −92)	<0.0001	−185 (−370,1)	0.05	−349 (−398, −299)	<0.0001	−749 (−927, −571)	<0.0001
Sugar↓ ^3,4^	3 (−44,51)	0.89	79 (−37,195)	0.18	−34 (−73,5)	0.08	−125 (−275,26)	0.10

1 2-levels (individuals within centers) linear mixed models adjusted for age, energy from non alcohol source, energy from alcohol, initial BMI, smoking status, education, physical activity, follow-up-time and plausible total energy intake reporting according to Goldberg (fixed effects). Intercept and macronutrient slope were entered as random effects. ^2^ Calibrated dietary data were obtained from country- and sex-specific calibration models. The 24-hour dietary values were regressed on the dietary values obtained from the main dietary questionnaire, adjusting for age, BMI at baseline total energy from non alcohol sources, energy from alcohol sources and study center. The sampling distribution of days and seasons of 24-hour dietary recall administration was corrected using a set of weights to reproduce an even distribution of recalls across weekday and season. The standard error of the coefficient was estimated using bootstrap sampling (10 loops). ^3^ Further adjusted for the percentage of protein. ^4^ Further adjusted for the percentage of fat. ^5^ Further adjusted for the percentage of carbohydrates. ^6^ Further adjusted for the percentage of plant fat. ^7^ Further adjusted for the percentage of unknown fat. ^8^ Further adjusted for the percentage of animal fat. ^9^ Further adjusted for the percentage of plant protein. ^10^ Further adjusted for the percentage of unknown protein. ^11^ Further adjusted for the percentage of animal protein. ^12^ Further adjusted for the percentage of starch. ^13^ Further adjusted for the percentage of sugar.

We evaluated whether the effect associated with a change in the carbohydrates proportion differed according to the glycemic index of the diet. A significant interaction according to the glycemic index median was observed for the association between weight gain and the substitution of 5% of energy from carbohydrates by 5% of energy from fat in women only (p for interaction = 0.46 in men and 0.03 in women, using uncalibrated data). However, associations were not statistically significant in both groups (β (95% CI) = −3 (−49,43), in women below the median and −41 (−88,6) in women above the median). A significant interaction according to the glycemic index median was also observed in women only for the association between weight gain and the substitution of 5% of energy from carbohydrates by 5% of energy from protein (p for interaction = 0.83 in men and <0.0001 in women). The association was positively significant in both group but stronger in women with low glycemic index (β (95% CI) = 474 (375,573) vs. 265 (163,366)).

We also evaluated whether the effect associated with a change in the carbohydrates proportion differed according to the fiber intake of the diet. No interaction was observed for the association between weight gain and the substitution of energy from carbohydrates by energy from fat in men (p for interaction = 0.42). However, a 5% higher proportion of fat at the expense of carbohydrates was negatively associated with weight change in women below (β (95% CI) = −50 (−95,-4)) but not above (17 (−29,62)) the fiber intake median (p for interaction<0.0001). In addition, a 5% higher proportion of protein at the expense of carbohydrates was associated with a higher weight gain in participants below as well as above the fiber intake median but the association was stronger in participants with high fiber intake (in men: β (95% CI) = 312 (206,418) vs. 192 (93,292), p for interaction = 0.03; in women: 446 (349,544) vs. 348 (254,443), p for interaction = 0.002).

The positive association between protein intake and weight change was observed in all age, BMI, smoking status, educational attainment, physical activity, dietary pattern and Goldberg criterion categories although significant interactions were observed according to BMI, smoking status, age, “prudent” dietary pattern and Goldberg criterion categories (Table 3). The association between percentage of energy from protein and weight change was slightly stronger in overweight, former smokers, participants ≥60 years old and participants in the second and third tertiles of the “prudent” dietary pattern. Positive significant associations were observed in all centers except Malmo (Sweden) were a positive association close to significance was observed (β (95% CI) = 127 (−3,257), p = 0.06).

**Table 3:** Adjusted 5y weight change (in g/5y) for the substitution of 5% of either fat or carbohydrates by 5% of protein according to age, smoking status, initial BMI, educational attainment, physical activity, “prudent” dietary pattern, Goldberg criterion and center.

	β (95% CI)	P	P for interaction
Age 1,2			0.0002
<60 years old	361 (266, 455)	<0.0001	
≥60 years old	491 (383, 600)	<0.0001	
Smoking status^1,2^			<0.0001
Never	368 (271, 465)	<0.0001	
Former	510 (407, 613)	<0.0001	
Current	329 (223, 435)	<0.0001	
Body Mass Index^1,2^			<0.0001
<25 kg/m^2^	294 (191, 396)	<0.0001	
25–29.9 kg/m^2^	531 (427, 635)	<0.0001	
≥30 kg/m^2^	345 (229, 460)	<0.0001	
Educational attainment 1,2			0.61
Less than an university degree	388 (292, 484)	<0.0001	
At least an university degree	371 (264, 479)	<0.0001	
			0.18
Physical activity 1,2			
Physically inactive or moderately inactive	407 (299, 515)	<0.0001	
Physically active or moderately active	448 (336, 559)	<0.0001	
Tertiles of the “prudent” dietary pattern score 1,2			<0.0001
1	193 (89, 296)	0.0003	
2	420 (322, 518)	<0.0001	
3	497 (397, 598)	<0.0001	
Goldberg criterion 1,2			<0.0001
Under reported	470 (365,574)	<0.0001	
Well reported	361 (261,462)	<0.0001	
Over reported	252 (98,405)	0.001	
Center 3			
France	593 (520,666)	<0.0001	
Spain	484 (380, 587)	<0.0001	
Italy	341 (264, 418)	<0.0001	
UK Cambridge	505 (357, 653)	<0.0001	
UK Oxford Health	410 (322, 497)	<0.0001	
UK Oxford general	347 (133, 560)	0.001	
NL Doetinchem	347 (64,630)	0.02	
NL Amsterdam/Maastricht	415 (232, 598)	<0.0001	
NL Utrecht	575 (411, 740)	<0.0001	
Greece	177 (23, 331)	0.02	
DE Heidelberg	360 (160, 561)	0.0004	
DE Potsdam	201 (99, 303)	0.0001	
SE Malmo	127 (−3, 257)	0.06	
SE Umea	167 (33, 301)	0.01	
Denmark	149 (68, 231)	0.0003	
Norway	222 (125, 320)	<0.0001	

Using uncalibrated data.

1 2-levels (individuals within centers) linear mixed models adjusted for sex, age, energy from non alcohol source, energy from alcohol, initial BMI, smoking status, education, physical activity, follow-up-time and plausible total energy intake reporting according to Goldberg (fixed effects). Intercept and protein intake slope were entered as random effects.

2 Potential effect modification was explored with the inclusion of interaction terms between each variable and protein intake in the models.

3 Center-specific associations were investigated using Generalized Linear Models adjusted for sex, age, energy from non alcohol source, energy from alcohol, initial BMI, smoking status, education, physical activity, follow-up-time and plausible total energy intake reporting according to Goldberg.

UK: United Kingdom; NL: The Netherlands; DE: Germany; SE: Sweden.

Adjusted relative risks (95% CI) of the risk of becoming overweight or obese after 5 years according to energy intake from protein and initial BMI are presented in Table 4. At baseline, 191,748 subjects were normal weight, 132,266 were overweight and 47,832 were obese. After 5 years, 41,842 (21.8%) normal weight subjects became overweight or obese, 21,213 (16%) overweight subjects became obese or morbidly obese, and 1,315 (2.8%) obese subjects became morbidly obese. Compared to diets with no more than 14% of energy from protein, diets with more than 22% of energy from protein were associated with a 24% (19,28%) higher risk of becoming overweight or obese in normal weight subjects at baseline, a 23% (17,30%) higher risk of becoming obese or morbidly obese in overweight subjects at baseline and a 26% (0,60%) higher risk of becoming morbidly obese in obese subjects at baseline. Dose-response associations were observed in normal weight and overweight participants (both P for trend <0.0001) but not in obese participants (P = 0.12).

**Table 4:** Adjusted Relative Risks (RR) [95% CI] of the risk of becoming overweight, obese or morbidly obese after 5 years 1 according to the percentage of energy from protein and the initial Body Mass Index (BMI).^2^

Percentage energy from protein 3	BMI <25kg/m^2^ at baseline N = 191,748			25≤ BMI <30kg/m^2^ at baseline N = 132,266			30≤ BMI <40kg/m^2^ at baseline N = 47,832		
	N (%)	N overweight or obese (%)	RR of the risk of becoming overweight or obese (95% CI)	N (%)	% obese or morbidly obese	RR of the risk of becoming obese or morbidly obese (95% CI)	N (%)	% morbidly obese	RR of the risk of becoming morbidly obese (95% CI)
≤14%	34,487 (18.0)	6,919 (20.1)	1	18,414 (13.9)	2,724 (14.8)	1	5,312 (11.1)	117 (2.2)	1
14.1–16%	48,529 (25.3)	9,877 (20.4)	0.99 (0.97, 1.01)	30,309 (22.9)	4,426 (14.6)	0.97 (0.93, 1.01)	10,106 (21.1)	265 (2.6)	1.17 (0.96, 1.44)
16.1–18%	51,379 (26.8)	10,789 (21.0)	1.01 (0.98, 1.03)	34,454 (26.1)	5,227 (15.2)	1.01 (0.97, 1.05)	11,920 (24.9)	281 (2.4)	0.98 (0.80, 1.21)
18.1–20%	34,092 (17.8)	7,809 (22.9)	1.05 (1.02, 1.08)	26,134 (19.8)	4,215 (16.1)	1.04 (0.99, 1.08)	9,815 (20.5)	265 (2.7)	1.03 (0.83, 1.27)
20.1–22%	15,390 (8.0)	4,049 (26.3)	1.14 (1.10, 1.17)	14,212 (10.8)	2,680 (18.9)	1.14 (1.08, 1.19)	6,035 (12.6)	188 (3.1)	1.11 (0.88, 1.40)
>22%	7,871 (4.1)	2,299 (30.5)	1.24 (1.19, 1.28)	8,743 (6.6)	1,941 (22.2)	1.23 (1.17, 1.30)	4,644 (9.7)	199 (4.3)	1.26 (0.99, 1.60)
P for trend			<0.0001			<0.0001			0.12

1 The BMI after 5-y was calculated from the 5-y weight change and their baseline height. The modified Poisson regression approach of Zou [32] was used to calculate the RR adjusted for sex, age, energy from non alcohol source, energy from alcohol, initial BMI, smoking status, education, physical activity, follow-up time, plausible total energy intake reporting according to Goldberg and center.

2 Subjects morbidly obese at baseline were excluded (n = 1,957).

3 Using uncalibrated data. We used the high-protein diet cut-off point from the American Diabetes Association (>20% of energy from protein) and further categorized participants by 2% increases.

## Discussion

In the present study investigating the relationship between the macronutrient composition of the usual diet and long term weight change in a large European cohort, a 5% higher proportion of protein, at the expense of either fat or carbohydrates, was positively associated with weight gain after 5 years. This association was observed in men and women, in normal weight as well as overweight and obese subjects and in all participating centers. A 5% higher proportion of fat at the expense of carbohydrates was not significantly associated with weight change. Compared to diets with no more than 14% of energy from protein, diets with more than 22% of energy from protein were associated with a 23-24% higher risk of becoming overweight or obese in normal weight and overweight subjects at baseline.

Low carbohydrate/high protein diets have been shown to promote weight loss in obese subjects in short term intervention studies [15]–[17]. However, the six longer term intervention studies have shown mixed results [33]–[38]. Four studies observed a higher weight loss after 6 months when carbohydrate intake was reduced [34]–[36], [38] but in two of them, the beneficial effect disappeared after 1 or 2 years [34], [35]. The two other studies did not find any beneficial effect at any time of the follow-up [33], [37]. A study suggested that the satiating effect of dietary protein varies inversely with habitual protein intake [39]. Therefore, the beneficial effect of high protein diet on satiety [40] could vanish when maintained during an extended period of time because of complete habituation to the increased protein intake.

Observational studies follow subjects during a longer time than intervention studies and have found inconsistent results so far [9]. In agreement with a recent review [9], we did not find any association between the iso-energetic replacement of energy from carbohydrates by energy from fat and weight change. However, we found a positive association between an increased proportion of protein in the diet and weight gain after 5 years. Our results on macronutrient composition are consistent with a recent study from a sub-sample of the EPIC cohorts investigating the total fat intake [41] or total protein intake [11] specifically. In a recent study among American men followed up during 7 years, the risk of obesity increased with increased percentage of energy from animal protein but decreased with increased percentage of energy from vegetable protein [10]. The risk of overweight was positively associated with the percentage of energy from animal protein but no significant association was observed with the percentage of energy from vegetable protein [10]. Other evidence reported that consuming a low-carbohydrate/high-animal protein diet was associated with higher all-cause mortality, whereas a low-carbohydrate/high-vegetable protein diet was associated with lower all-cause and cardiovascular disease mortality rates after 26 years of follow-up [42]. Both studies did not mutually adjust sources of protein. In agreement with those previous findings, weight change was positively associated with an increase in the proportion of animal protein but not with an increase in the proportion of vegetable protein in the non-mutually adjusted model. However, we observed positive associations for both sources of protein when sources of protein were mutually adjusted. This finding will need to be explored further in other populations.

The mechanism underlying the positive association between protein intake and weight gain is unclear. We previously shown that meat intake was positively associated with weigh gain [21] and methionine, an essential sulphur-containing amino acid mainly ingested in animal-derived foods, has been associated with increased BMI in a prospective cohort [43]. In addition, some experimental studies in mice suggested that macronutrient composition could play a role in the hypothalamic release of hormone affecting food intake. First, nutrient mixtures dominated by glucose could suppress the hypothalamic orexin/hypocretin system, which promotes reward seeking and food consumption, while nutrient mixtures dominated by amino acids would increase its activity [44]. Second, the decrease of food intake associated with protein-enriched diet could be counterbalanced by the hypothalamic melanocortin system to defend the body against weight variation [45]. Such mechanisms need to be further explored.

In our study, a 5% higher proportion of fat at the expense of carbohydrates was not significantly associated with weight change for participants both below and above the glycemic index median. This is in agreement with a previous 1-y controlled trial in type 2 diabetes patients showing no significant weight change difference between three different diets with various glycemic index, carbohydrate and fat amounts [46]. A 5% higher proportion of protein at the expense of carbohydrates was positively associated with weight change for participants above and even more for participants below the glycemic index median. Our study also reported a higher weight gain when protein was increased at the expense of carbohydrates rich in fibre compared to carbohydrates poor in fibre. All together, these results suggest that a high protein intake is more likely to lead to weight gain when consumed at the expense of good quality carbohydrates compared to poor quality carbohydrates.

The main strengths of the present study are the very large sample size and the high heterogeneity of macronutrients intake and obesity prevalence in the study population. We also partially corrected for measurement error of diet [30] and results were not modified.

However, several limits must be mentioned. First, misreporting may have influenced our results. A previous cross-sectional EPIC study showed that reported energy-adjusted protein intake did not differ across BMI categories whereas nitrogen excretion was significantly higher in obese participants compared to normal weight participants [47]. In addition, weight at follow-up was self-reported in most centers and might be underestimated, especially for overweight and obese participants [22]. A 5% higher proportion of protein, at the expense of either fat or carbohydrates was associated with a higher weight gain in overweight and obese as well as in normal weight participants, less likely to misreport their weight and diet [22], [48]. The accuracy of self-reported weight was improved with the use of a prediction equation [22] and results in the two centers with measured weights (Cambridge and Doetinchem) were in agreement with the rest of the cohort. This indicates that misreporting of weight at follow-up is most unlikely to explain our findings. Second, we were not able to consider change in diet before or during follow-up. We conducted sensitivity analyses with the exclusion of those likely to modify the diet because of previous illness, and the associations persisted. However, dieters who did not report any previous illness could not be excluded. High protein diets are a usual weight loss strategy in American populations [49] and dieters could also be more frequent in the high protein diet groups than in the low protein diet groups in our European population. Weight cycling and weight loss have been shown to be the strongest predictor of subsequent large weight gain in men and women respectively [50]. Therefore, we cannot rule out that the higher weight gains observed with the high protein diets are linked to weight loss failure and not to the protein intake of the diet *per se*. Third, we used BMI as an indicator of adiposity which is less precise than abdominal obesity measurement such as waist circumference or body composition measured by dual energy x-ray absorptiometry. Fourth, measurement error is likely to have attenuated the observed associations. Using urinary nitrogen excretion as a reference biomarker for protein intake, Kipnis et al have shown that even after calibration using the 24-hour dietary recall (as in the present analysis), the association between protein intake and disease could still be underestimated by up to 240% [51]. Finally, these results have been observed in a general European population and cannot be generalized to specific groups of individuals such as elderly [52] and pregnant women [53] for which beneficial effect of adapted protein intake have been suggested.

In agreement with other European data [54], the average percentage of energy coming from protein in our population exceeded the current WHO recommendation (10–15%) [55]. We showed a significant higher risk of becoming overweight or obese from 18% of energy from protein in normal weight subjects and from 20% of energy from protein in overweight subjects. These estimates could be higher with a longer follow-up and are likely to be greatly underestimated compared to an estimate obtained using urinary nitrogen excretion as a reference biomarker for protein intake [51]. These results show that consuming an amount of protein above the recommendation may be deleterious for weight maintenance through adult life. Confirmation in other large scale cohort studies is warranted. In addition, the mechanisms by which habitual diets characterized by a sustained high proportion of protein lead to long term weight gain deserves further investigations.
